# Canine Pluripotent Stem Cells: Are They Ready for Clinical Applications?

**DOI:** 10.3389/fvets.2015.00041

**Published:** 2015-10-07

**Authors:** Dean H. Betts, Ian C. Tobias

**Affiliations:** ^1^Department of Physiology and Pharmacology, Schulich School of Medicine & Dentistry, The University of Western Ontario, London, ON, Canada; ^2^Children’s Health Research Institute, Lawson Health Research Institute, London, ON, Canada

**Keywords:** canine, dog, embryonic stem cells, genome editing, induced pluripotent stem cells, transplantation therapy

## Abstract

The derivation of canine embryonic stem cells and generation of canine-induced pluripotent stem cells are significant achievements that have unlocked the potential for developing novel cell-based disease models, drug discovery platforms, and transplantation therapies in the dog. A progression from concept to cure in this clinically relevant companion animal will not only help our canine patients but also help advance human regenerative medicine. Nevertheless, many issues remain to be resolved before pluripotent cells can be used clinically in a safe and reproducible manner.

## Canine Embryonic Stem Cells

We were among a number of research groups between 2007 and 2009 who derived numerous canine embryonic stem cell (cESC) lines from blastocyst stage embryos (1–6) (Table 1). These cESC lines expressed the core pluripotency markers (Oct4, Sox2, and Nanog) and were capable of differentiating into representative lineages of all three germ layers *in vitro*. Limited proliferative potential was observed for several cESC lines tested by a few research groups (1, 2), while only the cESC lines established by the Hough and Betts labs were capable of forming modest teratomas *in vivo* (4, 5).

**Table 1:** **Summary of canine embryonic stem cells (cESCs) derived from dog embryos**.

Reference	Derivation method	Basal media	Growth factors	Feeder layers	*In vitro* differentiation	*In vivo* differentiation	Long-term culture
(1)	Mechanical	DMEM/F12, 20% FBS	mLIF	MEFs (mitomycin C)	By morphology	ND	Two cell lines, eight passages
(2)	Explant	DMEM/F12, 15% FBS	hLIF	MEFs	Flow cytometry/RT-PCR	ND	One cell line, ND
(3)	Explant	DMEM/F12, 15% FBS	hLIF	MEFs (γ-irradiation)	EB formation, differentiation markers	Unsuccessful NOD/SCID	One cell line, >20 passages
(4)	Explant	DMEM/F12, 15% ESC- qualified FBS or KSR	hLIF, hbFGF	MEFs (γ-irradiation)	EB formation, directed differentiation	Teratomas NOD/SCID	Four ESC lines, >25 passages
(5, 6)	Explant and immuno-dissection	KO-DMEM, 15% KSR	hLIF, hbFGF	MEFs (γ-irradiation)	EB formation, directed diff. functional test	Limited teratomas NOD/SCID	10+ cESC lines, >37 passages

*ND, not determined; KO, knockout; KSR, knockout serum replacement*.

Canine ESC colonies seem to exhibit two phenotypically discrete morphologies, with some colonies having distinct borders and a flattened appearance similar to human ESCs (hESCs), whereas others were three-dimensional, round, tightly packed colonies that resemble mouse ESCs (mESCs). Interestingly, our mESC-like cESCs could not be successfully propagated long-term in KnockOut serum (KSR)-containing medium supplemented with leukemia inhibitory factor (LIF) and basic fibroblast growth factor (bFGF) (5), while the cESCs derived by Hayes et al. could not be cultured long-term in medium containing LIF only, but formed loosely adhered colonies interspersed with feeder cells (3). The hESC-like cESCs generated differed in growth factor dependency, as prolonged maintenance required supplementation with either soley LIF or LIF and bFGF (4, 5). Morphologically distinct cESC may represent multiple and perhaps metastable pluripotent states (e.g., naïve and primed) that have been recently characterized for both mouse and human pluripotent stem cells (PSCs) (7–9). It appears as though naïve and primed pluripotency may be at least partly conserved within other Eutherian mammals because PSC lines with naïve-like characteristics have been derived from rabbits (10), pigs (11), cows (12), and perhaps the dog (13). Many of these lines must be established and/or maintained in the presence of LIF and inhibitors of glycogen synthase kinase 3β and the MAP kinase pathway (2i), which suppress pro-differentiation stimuli (14). In depth “omics” examination of the molecular signatures underlying cESCs and preimplantation dog embryos will allow a greater understanding of the most relevant embryonic counterparts of differing metastable pluripotent states and will help define and optimize specific culture conditions for their unlimited self-renewal and directed differentiation into therapeutically desirable cell types.

## Canine-Induced Pluripotent Stem Cells

In 2006, Takahashi and Yamanaka (15) demonstrated that mouse embryonic fibroblasts could be reprogramed to a pluripotent state by the over-expression of exogenous pluripotency transcription factors (Oct-4, Klf4, Sox2) and c-Myc (OKSM). These so-called induced pluripotent cells (iPSCs) exhibit indefinite proliferative capacity and were deemed pluripotent by the expression of ESC-specific genes and the formation of embryoid bodies and teratomas. It was also shown that they could contribute to chimeric embryos with germline competency, demonstrating that iPSCs and ESCs exhibit similar *in vivo* differentiation capabilities (16). This initial publication has spurred a whole new research field and subsequently iPSCs have now been generated in a number of different mammals including the dog (17–19). However, random infection and integration of viral transgenes introduces both population heterogeneity and asynchrony to iPSC generation. Presumptive iPSC clones should be extensively screened with surface markers that best correlate with active endogenous pluripotency circuitry (20, 21). Unfortunately, there are discrepancies with regards to the antigen profile of ciPSCs and not all laboratories have access to canine embryos to properly control for antibody specificity (22–24). To date, there have been seven reports on the production of canine (c)iPSCs (Table 2). Two studies reprogramed canine embryonic fibroblasts (23, 25), while three used adult skin fibroblasts of various ages as the source of parental cells (22, 26, 27) by either retro- or lentiviral transduction of dog (25), human (23, 26, 27), or mouse (22) pluripotency transcription factor homologs. Two studies reported the generation of ciPSCs from adipose multipotent stromal cells (24, 26), whereas the Cibelli laboratory reprogramed from testicular fibroblasts (28). The ability to reprogram cells from multiple tissues accessible during routine procedures minimizes unnecessary harm to the patient, but it is currently unknown why different somatic cell types achieve dissimilar reprograming efficiencies (26).

**Table 2:** **Summary of canine induced pluripotent stem cells (ciPSCs) generated from dog cells**.

Reference	Cell sources	Basal media	Media supplements	Feeder layers	*In vitro* differentiation	*In vivo* differentiation	Reprogram method
(25)	Embryonic fibroblasts	Primate ES medium	bFGF, hLIF + 3i VPA	MEFs	*In vitro* differentiation	ND	Retrovirus (canine OKSM)
(28)	Testicular fibroblasts	DMEM/F12, 15% KSR	bFGF, hLIF	MEFs	Embryoid bodies	No teratoma formation	Lentivirus (human OKSM)
(26)	Adipose stromal cells, skin fibroblasts	KO-DMEM, 20% ES qualified FBS	bFGF, hLIF	MEFs	Embryoid bodies	Teratomas	Lentivirus (human OKSM)
(27)	Dermal fibroblasts	KO-DMEM/F12, 20% KSR	mLIF	MEFs	ND	Germ cell-like tumor	Lentivirus (human OKSMLN)
(22)	Skin fibroblasts	DMEM/F12, 20% KSR	bFGF, hLIF + 2i	MEFs	Embryoid bodies	Teratomas	Retrovirus (mouse OKSM)
(23)	Embryonic fibroblasts	DMEM/F12, 20% KSR	bFGF, hLIF	MEFs	Embryoid bodies, platelets	ND	Lentivirus (human OKSM)
(24)	Ad-MSCs	DMEM/F12, 15% FCS	bFGF, LIF	MEFs	Spontaneous differentiation, embryoid bodies	ND	Retroviral (human OKSM)

*ND, not determined; OKSM, OCT4, KLF4, SOX2, and c-MYC transgenes; LN, LIN28, NANOG transgenes*.

Like their cESC counterparts, the majority of generated ciPSCs favor dual-factor culture of both bFGF and LIF for proliferation in the undifferentiated state (22–26, 28), but with only a few reports of *in vivo* teratoma formation (22, 26). A LIF-dependent ciPSC line was established using the standard “Yamanaka” factors (*OCT4, KLF4, SOX2*, and *c-MYC*) plus *LIN28* and *NANOG*, however germ cell-like tumors were formed upon engraftment into NOD/SCID mice (27). It is presently unclear if species-specificity of reprograming factors is important for proper pluripotency induction in the dog because most of the ciPSCs were generated by retroviral transduction with human and murine homologous sequences that remain expressed in late passage ciPSCs (22, 23, 27, 28). However, these retroviral methodologies are not favorable for prospective clinical transplantation therapies because of their biased, non-random integration of transgenic sequences in promoter and coding regions, which result in dysregulation of endogenous gene expression leading to possible tumorigenesis and/or immunogenicity problems (29, 30). Alternatively, non-integrating reprograming systems have been developed and utilized in other species including episomal vectors, mini circle DNAs, plasmid vectors, small molecules, mRNAs, recombinant proteins, and transposons (31). Utilization of either the non-integrating Sendai viral-based or Cre-excisable lentiviral-based reprograming systems along with small molecular facilitators of DNA demethylation on canine fibroblasts should facilitate the generation of transgene integration-free iPS cells in the dog.

## Considerations for Clinical Translation of Canine Pluripotent Stem Cell Technologies

The generation of canine pluripotent stem cells (cPSC) with disease-specific alleles and the derivation of cell types afflicted by the disease promises the development of novel cellular disease models, drug screening platforms, and potential regenerative therapies. It seems that cPSC technologies may be on the verge of clinical translation in the dog (6, 23, 26, 32), however to facilitate these cPSC-based treatments a number of hurdles need to be overcome. Canine iPSC generation efficiencies have not been routinely included in publications, but have been reported as low as 7.0 × 10^−4^% (27). This poses a fundamental question regarding the optimal trophic and physicochemical requirements of cPSCs. To date, we are unsure of how or when key signaling pathways are critical for the growth and epigenetic resetting of transduced canine cells. Despite of this fact, two canine somatic cell reprograming protocols have applied the transforming growth factor β (TGFβ) receptor inhibitor A-83-01 (25, 27), presumably to facilitate epithelialization of transduced cells.

Current differentiation strategies for PSCs produce cell types that correspond to immature, but lineage-committed cells from embryonic or fetal sources. Subsequently, PSC derivatives are matured with sequential growth factor exposure within a microenvironmental niche that favors the desired cell type (33). We have established synaptically competent neurons from cESCs, but were only functional when co-cultured with primary neurons derived from canine fetuses (6). Nishimura and colleagues differentiated ciPSCs into mature megakaryocytes that released functional platelets *in vitro* (23), while Deanne Whitworth has recently derived highly proliferative mesenchymal stromal cells (MSCs) from ciPSCs that undergo robust differentiation into the osteo-, chondro- and adipogenic cell lineages (32). Excitingly, Joseph Wu’s group at Stanford has demonstrated the preclinical potential of ciPSCs by treating immunodeficient mouse models of myocardial infarction and hindlimb ischemia with transplanted endothelial cells derived from ciPSCs (26).

All cPSC lines have been established and differentiated in culture systems that expose the canine cells to proteins or feeder cells from another species. Xeno-contamination is an important biosafety concern for graft recipients and can be limited if chemically defined culture media (i.e., serum free with recombinant factors) can be adapted for the derivation and maintenance of cPSCs (34). Moreover, technologies that enable the characterization of desired cell fates in individual cells (e.g., flow-based quantification of marker expression, RNA-sequencing, chromatin state, etc.) and the production of highly purified cell types with adult-like functional properties *in vivo* (e.g., small molecule inhibitors/activators of signaling pathways, three-dimensional scaffolds, etc.) need to be further developed (33).

Combining patient-specific ciPSC generation with targeted genome editing technologies (35) will enable correction of genetic defects, thereby offering potential treatment of some inherited canine diseases (36). Initially, genome editing will allow for the genetic alteration of existing cESC lines or gene correction of ciPSC lines that have been reprogramed from diseased canine cells to produce isogenic comparisons that can be utilized for disease modeling. In the long term, diseased and allele-corrected ciPSCs will serve as provocative tools within pharmacological screening platforms for efficient and predictive drug discovery and toxicity studies for the treatment of various diseases in the dog (36, 37). It is anticipated over the next few years that the CRISPR/Cas9 genome editing system will be rapidly implemented as a means to conduct loss-of-function gene mutation studies in cESCs and/or to correct genetic mutations in ciPSCs generated from fibroblasts with a disease-causing allele, as a proof of principle study.

Realizing the therapeutic potential of PSCs for clinical applications remain a central goal for the veterinary and scientific communities. Since at least half of all heritable canine diseases are known to have human equivalents (38), the dog will increasingly become an unrivaled translational animal model for developing stem cell-based therapies in humans as well. Thus, key components for any successful cPSC-based treatment will be the ability to efficiently produce ciPSCs without off-target genetic alterations or karyotypic abnormalities (39), to produce a pure population of cPSC derivatives in a scalable and good manufacturing practice (GMP)-cooperative manner, and the selection of appropriate diseases to target for regenerative medicine in the dog (33, 36). The relatively relaxed legal and ethical regulation of veterinary stem cell research compared to human medicine has facilitated the development and clinical application of a number of unproven cell-based therapies in large animals including the dog (40). The Food and Drug Administration (FDA) recently issued a draft guidance for industry on cell-based products for animal use (Draft Guidance #218) that clarifies how existing FDA regulations apply to cell-based products and encourages stakeholders to communicate and interact with the FDA early in the developmental process for each stem cell product. These guidelines and future FDA regulations of veterinary stem cell-based therapies may spearhead the much-needed double-blinded, randomized, and controlled clinical trials to properly evaluate the safety, utility, and efficacy of cPSC-based therapies in the dog.

## Conflict of Interest Statement

The authors declare that the research was conducted in the absence of any commercial or financial relationships that could be construed as a potential conflict of interest.
